# Optic neuritis and meningoencephalitis associated with an ovarian teratoma in a dog

**DOI:** 10.1093/jvimsj/aalag088

**Published:** 2026-05-20

**Authors:** Hunter P Sapienza, Brian G Murphy, Kelly O’Connell, Nina Samuel, Sara A Adelman, Soohyun Kim, Karen M Vernau, Christine M Toedebusch, Adrien M Dupanloup

**Affiliations:** William R. Pritchard Veterinary Medical Teaching Hospital, Weill School of Veterinary Medicine, University of California, Davis, Davis, CA, United States; Department of Pathology, Microbiology and Immunology, Weill School of Veterinary Medicine, University of California, Davis, Davis, CA, United States; Sacramento Veterinary Referral Center, Sacramento, CA, United States; Philadelphia Animal Specialty and Emergency, Philadelphia, PA, United States; Vet Vision Center, Lambertville, NJ, United States; Department of Surgical and Radiological Sciences, Weill School of Veterinary Medicine, University of California, Davis, Davis, CA, United States; Department of Surgical and Radiological Sciences, Weill School of Veterinary Medicine, University of California, Davis, Davis, CA, United States; Department of Surgical and Radiological Sciences, Weill School of Veterinary Medicine, University of California, Davis, Davis, CA, United States; Department of Surgical and Radiological Sciences, Weill School of Veterinary Medicine, University of California, Davis, Davis, CA, United States

**Keywords:** immune-mediated, meningoencephalitis, neurology, ophthalmology, paraneoplastic, pathology

## Abstract

A 3-year-old intact female Pit Bull Terrier mix-breed dog was examined because of acute vision loss. Ophthalmologic examination was consistent with bilateral optic neuritis. Abnormalities were not detected on the remainder of the neurological examination. Brain MRI identified multifocal lesions involving the optic nerves and cortical gray matter, while cerebrospinal fluid analysis demonstrated mixed pleocytosis. Abdominal ultrasonography revealed a cystic ovarian mass. Ovariohysterectomy was performed and histopathologic examination was consistent with an ovarian teratoma. Neural tissue within the teratoma featured extensive perivascular cuffing and immunohistochemistry confirmed mixed lymphocytic infiltration. After tumor resection, the dog was treated with corticosteroid for 40 days, leading to resolution of pleocytosis. Vision was preserved in the left eye but there was persistent vision loss in the right eye. Three years after initial presentation, good quality of life with no behavioral evidence of neurological disease or relapse was reported. This case highlights a suspected paraneoplastic neurological syndrome associated with an ovarian teratoma.

## Graphical Abstract

**Graphical Abstract:**
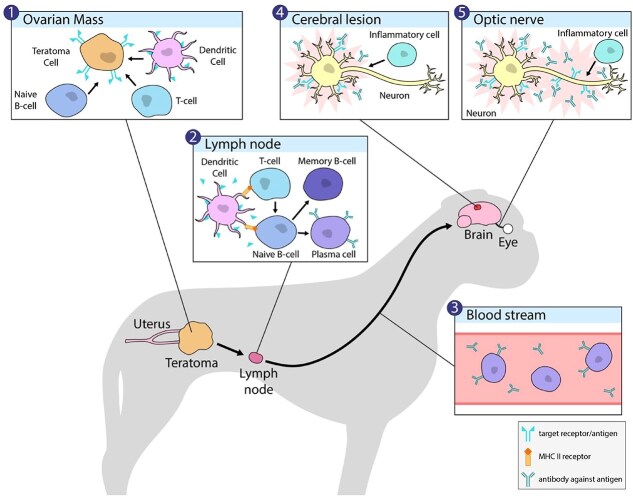

## Introduction

Ovarian teratomas are rare, typically benign tumors in dogs, characterized by the intralesional presence of multiple differentiated tissue types from all 3 germ layers and are presumed to be derived from multipotent germ cells.^1,2^ These tumors are generally well-differentiated and include a combination of ectodermal epithelium or neuroepithelium, mesodermal mesenchymal tissues, and endodermal gastrointestinal and/or respiratory mucosa. Hair, cartilage, teeth, and bone are often identifiable grossly and histologically. After surgical removal, animals with benign teratomas have an excellent prognosis.^1,2^

Optic neuritis refers to an array of diseases that cause acute vision loss secondary to demyelination of the optic nerve.^3^ Affected dogs typically present with acute blindness and funduscopic examination can reveal papillitis and neuroretinitis. Potential etiologies include immune-mediated, infectious, and neoplastic diseases, though many cases remain idiopathic.^3–6^ Optic neuritis is commonly diagnosed with a combination of neuro-ophthalmological examination, magnetic resonance imaging (MRI), and cerebrospinal fluid (CSF) analysis. Optic neuritis can be either localized to the optic nerve or associated with multifocal to diffuse brain lesions that often preferentially affect the white matter with optic nerve involvement.^3^ Granulomatous meningoencephalitis is the most common histopathologic diagnosis on postmortem examination of affected dogs, though neoplastic etiologies are also reported.^4^ More than 10% of dogs in a recent retrospective study were diagnosed with optic neuritis associated with primary optic nerve, orbital, or intracranial neoplasia.^4^

In humans, ovarian teratomas can be associated with paraneoplastic encephalitis and optic neuritis.^7–9^ Paraneoplastic neurological syndrome (PNNS) refers to autoimmune neurological dysfunction triggered by a tumor at a distant anatomic site when autoantibodies form against neural antigens produced by the neoplastic process or proteins expressed by intratumoral neural tissue.^7,9^ Anti-N-methyl-D-aspartate receptor (NMDAR) encephalitis is the most common PNNS associated with ovarian teratomas in human patients, although recent case reports also reveal an association with ovarian teratomas and neuromyelitis optica spectrum disorder, an autoimmune disorder that causes inflammation primarily in the optic nerves and spinal cord.^7–21^

Here we describe a case of acute optic neuritis and meningoencephalitis in a 3-year-old female intact Pit Bull Terrier mix-breed dog with an ovarian teratoma and suspected paraneoplastic meningoencephalitis. We present the proposed pathogenesis of paraneoplastic encephalitis and optic neuritis associated with the presence of an ovarian teratoma, and the clinical response after ovariohysterectomy.

## Materials and methods

The CF-1 Retinal Camera with a 50°-wide angle lens (Canon, Tokyo, Japan) was used to obtain color and red-free fundus photographs. All MRI studies were performed on a 1.5 T Signa MRI scanner (General Electric Medical Systems, Milwaukee, WI, USA). Cerebrospinal fluid was obtained via cerebellomedullary cisternal tap with a 25G 1.5-inch needle. After routine ovariohysterectomy, the uterus, ovaries, and associated mass were fixed in 10% buffered formalin for 48 h and the ovarian mass was decalcified in 15% formic acid. Immunohistochemistry (IHC) assays detecting antigens for CD3, CD79a, CD20, MUM1, and IBA1 were performed on 5-μm-thick, formalin-fixed, paraffin-embedded tissue sections using a streptavidin biotin detection system (Biocare Medical, Pacheco, CA, USA). Sections of normal canine lymph node tissue were used as positive controls for CD3, CD79a, CD20, MUM1, and IBA1 staining, with negative control slides lacking the primary antibody. Additional details regarding the IHC protocols for each assay are included in Table S1.

## Case description

A 3-year-old intact female Pit Bull Terrier mixed-breed dog was presented to the ophthalmology service at the University of California Davis Veterinary Medical Teaching Hospital (VMTH) for evaluation of acute blindness. Two days earlier, the owner noted that the dog was having difficulty seeing and exhibiting apparent pain. The dog was evaluated by a veterinary ophthalmologist who diagnosed optic neuritis bilaterally (OU, *oculus uterque*) and blindness in the right eye (OD, *oculus dexter*). CBC, biochemistry panel, and urinalysis revealed no relevant findings other than mild neutrophilia (14 990/uL, RI 2950-11 640/uL). The dog was referred to the VMTH for further evaluation. On neuro-ophthalmological examination at the VMTH, the dog was able to navigate the room normally. The menace response, dazzle reflex, and direct pupillary light reflex were absent OD and present in the left eye (OS, *oculus sinister*). Consensual pupillary light reflex OD to OS was absent, and consensual pupillary light reflex OS to OD was intact, consistent with a right-sided deficit of the optic nerve. Other cranial nerves were intact. A dilated funduscopic examination revealed bilaterally raised optic nerve heads with indistinct margins. Abnormalities were not detected on the remainder of the physical, neurological, and ophthalmological examinations.

### Diagnostic findings

**Figure 1:**
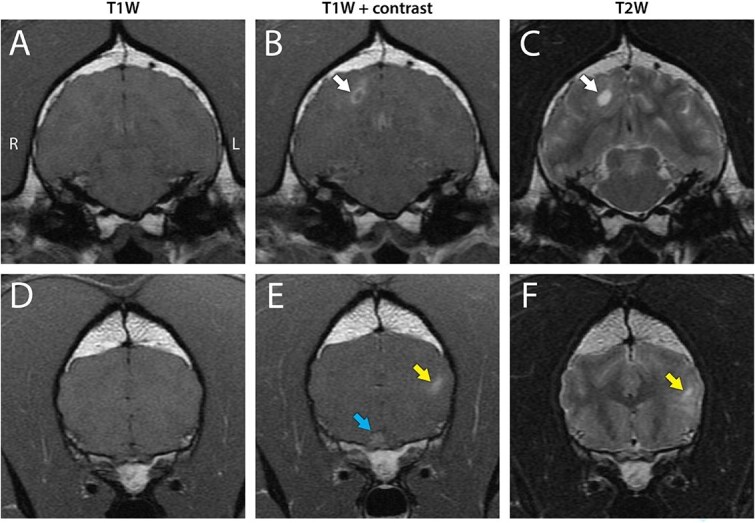
Transverse MR imaging revealed moderate bilateral optic neuritis and multifocal encephalitis with lesions throughout the left frontal and temporal lobes, right temporal lobe, right and left occipital lobes, and the spinal cord at the level of C2. All lesions were predominantly gray-matter-distributed and T2 hyperintense, and T1 isointense with contrast enhancement. (A–C) Right dorsal occipital lobe lesion (white arrows) with T2W hyperintensity and mild contrast enhancement. A small central fluid pocket was T2 hyperintense and failed to suppress on the FLAIR sequence. (D–F) Bilateral thickening and T1 contrast enhancement of the optic nerves with the right (blue arrow) more affected than the left. Left lateral temporal lobe lesion (yellow arrows) with mild contrast enhancement. Abbreviations: FLAIR = fluid-attenuated inversion recovery; MR = magnetic resonance.

Abdominal ultrasonography revealed a cystic and partially mineralized right ovarian mass. Cytology of the ovarian mass showed moderate neutrophilic inflammation with abundant cystic material and keratinized squamous epithelial cells. Brain MRI (Figure 1) demonstrated evidence of multifocal lesions throughout the left frontal and temporal lobes, right temporal lobe, right and left occipital lobe, and the spinal cord at the level of C2. Moderate bilateral thickening and contrast enhancement of the optic nerves were consistent with optic neuritis. The right optic nerve was affected throughout its entire length, with contrast enhancement and enlargement to the level of the optic chiasm. Mild medial retropharyngeal lymphadenomegaly was present bilaterally. Cisternal CSF analysis revealed mild mixed inflammatory pleocytosis (4 nucleated cells/uL [RI 0-3 cells/uL] characterized by 24% neutrophils, 33% small mononuclears, 43% large mononuclears; total protein: 39 mg/dL [RI < 25-30 mg/dL], and 25 red blood cells/uL). Abnormalities were not detected on repeat blood count and biochemistry panels. Testing for *Coccidioides* spp., *Neospora caninum, Toxoplasma gondii*, and *Brucella canis* antibodies, and *Cryptococcus* spp. antigen was negative.

### Therapeutic intervention

Ovariohysterectomy was performed. After recovery from surgery, prednisone (1 mg/kg by mouth twice a day) was prescribed. The dog was hospitalized for 2 days, during which time the neurological status remained static.

### Ovarian mass pathology

**Figure 2:**
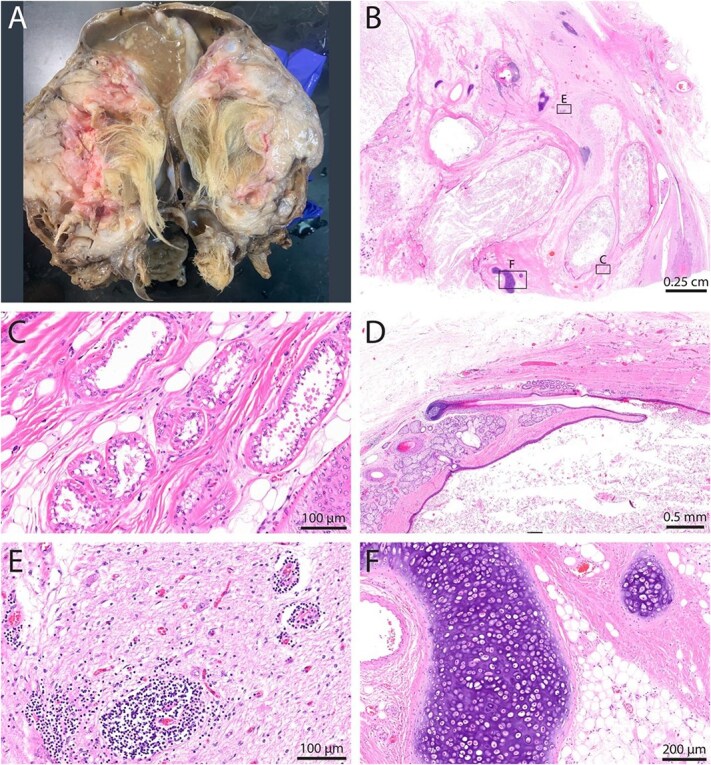
Gross pathology (A) and histopathology (B-F) of the ovarian mass confirmed a diagnosis of ovarian teratoma. Histologic examination of ovarian teratoma (B) revealed multiple tissue types presumed to be derived from pluripotent germinal tissue, including endoderm (C), ectoderm (D—from different cross-section than B), neuroectoderm (E), and mesoderm (F). Relevant features included ducts resembling endodermal alimentary or respiratory epithelium (C); foci of keratinized stratified squamous epithelium with mature hair follicles and well-differentiated cutaneous adnexa (D); neural tissue including neuropil, neurons, and glial cells, featuring perivascular cuffing with lymphocytic infiltration (E); aggregates of adipose and skeletal muscle with strips of differentiated hyaline cartilage (F). (H&E-stained tissues). Abbreviation: H&E = hematoxylin and eosin.

The left ovary and uterus were grossly within normal limits. The right ovary measured 14 × 9 × 4 cm with multiple lobulated masses distorting the surface. On section, the ovarian lesion featured numerous cavitations and was variably composed of pale brown fluid, hair, and small fragments of firm, white, smooth material that was easily removed and difficult to cut (bone and cartilage) (Figure 2A). A single recognizable tooth-like structure was expelled while trimming.

**Figure 3:**
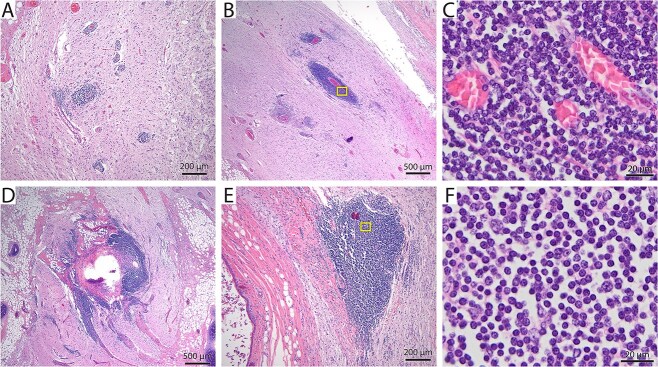
H&E staining of cross-sections from the ovarian teratoma displayed neural tissue with perivascular cuffing (A-C) and foci of lymphocytic inflammation (D-F). Abbreviation: H&E = hematoxylin and eosin.

**Figure 4:**
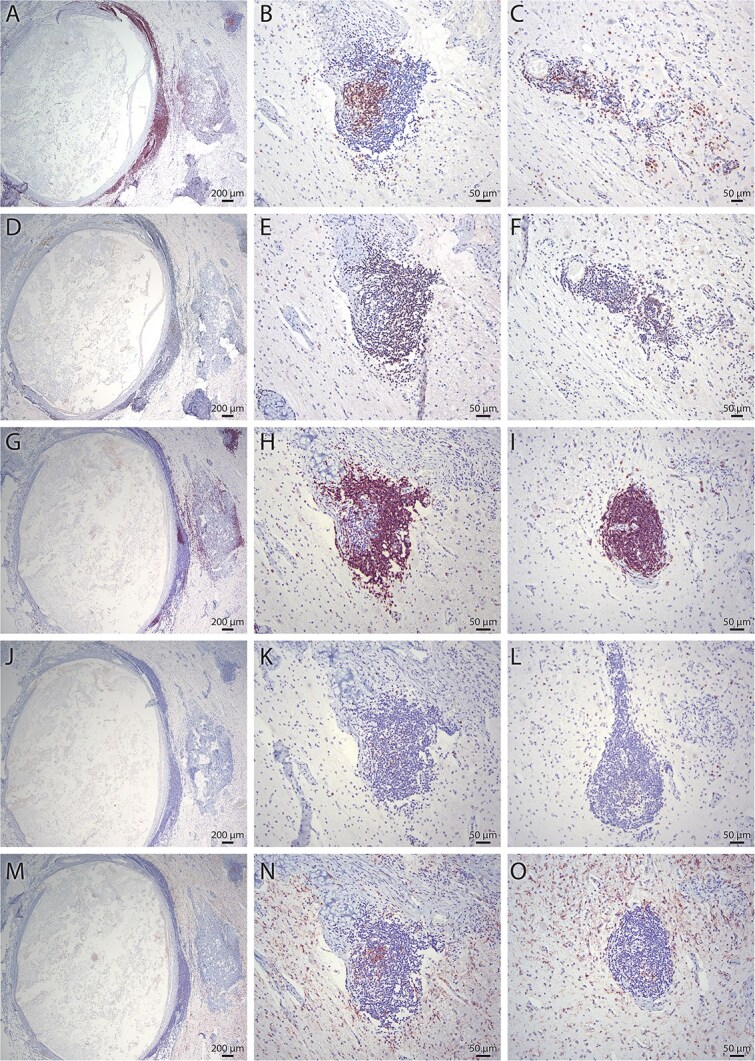
Immunohistochemistry of neural tissue within the ovarian teratoma characterized perivascular cuffing as mixed lymphocytic infiltration of predominantly CD3 + T cells (A-C) with some CD79a + B cells (D-F) and CD20 + B cells (G-I). A few MUM1 + plasma cells (J-L) and IBA + histiocytes (M-O) were present within the inflammatory foci.

On histologic examination, the right ovarian mass was heterogeneously characterized by multiple intermixed, well-differentiated and mature ectodermal, endodermal, and mesodermal tissues suspected to be derived from pluripotent germinal cells, consistent with an ovarian teratoma (Figure 2). Aggregates of adipose connective tissue and skeletal muscle tissue loosely encircled multiple foci of keratinized stratified squamous epithelium with mature hair follicles and well-differentiated cutaneous adnexa (Figure 2D). One examined section featured neural tissue including neuropil, neurons, and glial cells (Figure 2E). A section of neuroparenchyma had a region of ependymal cells lining a ventricle-like space with associated rests. Neural tissue also featured extensive perivascular cuffing with lymphocytic infiltration, predominantly comprising CD3 + T cells and some CD79a + B cells (immunohistochemistry assays, Figures 3 and 4, suspected pathogenesis, Figure 6). Scattered throughout the ovarian mass were strips of differentiated hyaline cartilage, ducts resembling endodermal alimentary or respiratory mucosa, an island of poorly mineralized bone, and multifocal regions of cardiac muscle (Figure 2C and F). One examined section of the left ovary contained multiple follicles of varying sizes, and a section of uterus revealed no abnormalities.

### Recheck examination and follow-up

**Figure 5:**
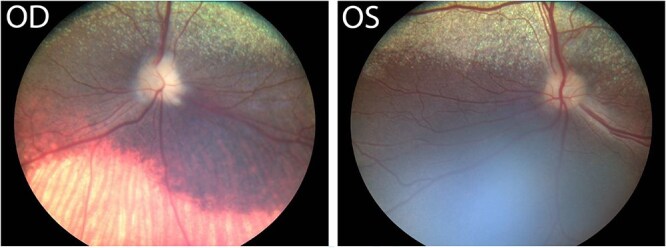
Bilateral fundoscopy images at the post-operative examination show that optic nerve head swelling with indistinct margins OU had resolved. The fundic exam was consistent with optic nerve atrophy secondary to previous optic neuritis with a rounded, pale optic nerve head and subjectively thinned myelin. Otherwise, the retinal vasculature and the tapetal and non-tapetal fundus were normal. Note: Normal choroidal vessels are visible in the non-tapetal fundus in the right eye, which is a normal variation (OD, right eye; OS, left eye). Abbreviations: OD = *oculus dexter*; OS = *oculus sinister*; OU = *oculus uterque*.

The dog was re-evaluated 3 weeks after discharge. On neuro-ophthalmologic examination, the dog was blind OD with absent menace response, dazzle reflex, and direct pupillary light reflex. The menace response and direct pupillary light reflex were intact OS. Bilateral fundoscopic examination performed at this time was consistent with optic nerve atrophy secondary to previous optic neuritis with a rounded, pale optic nerve head and subjectively thinned myelin (Figure 5). Fundoscopic images from the initial visit were unavailable for comparison, as these images are not routinely archived. Abnormalities were not detected on the remainder of the neurological and ophthalmological exams.

**Figure 6:**
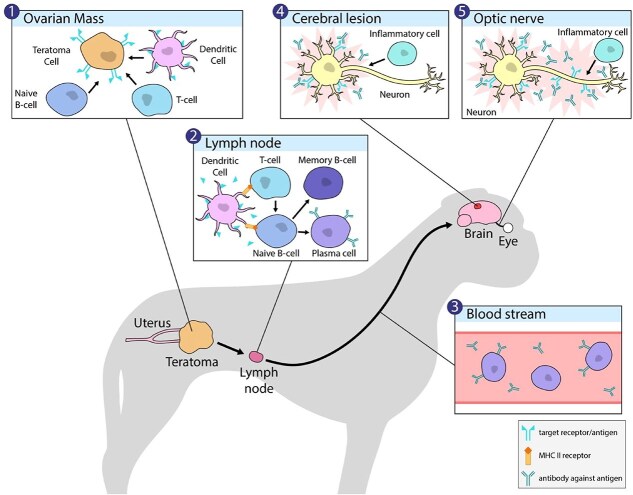
Proposed pathogenesis of paraneoplastic meningoencephalitis and optic neuritis associated with the presence of an ovarian teratoma. (1) The immune response to ovarian teratoma cells is characterized by lymphocytic inflammation and detection of intratumoral neural tissue antigen as foreign by dendritic cells; (2) antigen presentation in regional lymph nodes by dendritic cells leads to activation of T-cells and naïve B-cells that avoid elimination by central and peripheral tolerance. Naïve B-cells differentiate into memory B-cells and plasma cells, which generate autoantibodies against neural tissue antigen; (3) plasma cells and antibodies travel systemically, and antibodies migrate to the brain; (4 and 5) autoantibodies target antigen found on neural proteins and receptors found in the brain and optic nerve, leading to autoimmune-mediated cerebral lesions and optic neuritis.

The dog was anesthetized and cisternal CSF was collected. Cerebrospinal fluid analysis was nearly acellular and within normal limits (1 nucleated cell/uL [RI 0-3 cells/uL] characterized by rare small and large mononuclear cells; total protein: 19 mg/dL [RI < 25-30 mg/dL]; < 1 red blood cell/uL). Prednisone was continued (1 mg/kg by mouth twice a day) for 20 days, then discontinued by the owner without a tapering dose, resulting in a 40-day course of corticosteroid treatment.

Upon phone consultation 3 years after initial presentation, the owner reported a static clinical status and good quality of life. The owner noted persistent visual deficits that were more conspicuous at night, but no signs of relapse, progressive vision loss, or new deficits over the 3-year period.

## Discussion

In this case study, we described a suspected paraneoplastic syndrome associated with an ovarian teratoma in a dog with optic neuritis and meningoencephalitis. Histopathology of the teratoma revealed intratumoral neural tissue infiltrated by lymphocytes, supporting an antigen-driven immune activation. Aberrant presentation of neural antigens likely triggers B- and T-cell responses, resulting in autoantibody production, immune-mediated targeting against central nervous system antigens, and subsequent neurological deficits. Treatment involved removal of the ovarian teratoma and immunosuppressive therapy.

Ovarian teratomas are a well-defined cause of paraneoplastic neurologic syndromes in the human medical literature.^7,8^ Confirmatory testing requires autoantibody detection in CSF, although CSF pleocytosis and detection of an ovarian teratoma coupled with signs of neurological disease can be indicative of an immune-mediated paraneoplastic syndrome.^8^ Early detection and treatment are associated with a good prognosis.^7,11^

Anti-NMDAR encephalitis is the most often reported autoimmunity associated with ovarian teratomas, although cases of paraneoplastic meningoencephalitis with involvement of the optic nerve are also described due to the formation of autoantibodies directed against myelin oligodendrocyte glycoprotein (MOG) and aquaporin-4 (AQP4).^7,17–22^ Across published series, ovarian teratomas have been reported in approximately 37.4% of anti-NMDAR encephalitis cases, with individual studies reporting an incidence between 20% and 45%.^8,13,23^ Ovarian teratomas account for at least 90% of neoplasms responsible for paraneoplastic anti-NMDAR encephalitis.^11,23^ However, less than 2% of ovarian teratomas result in a PNNS.^7,14,16^ Ovarian teratomas are estimated to comprise only 2% of identified ovarian tumors in dogs, compared to approximately 20% of ovarian tumors in humans.^1,7^

The proposed mechanism for these paraneoplastic syndromes involves the apoptosis of neoplastic neural cells within the teratoma, followed by a lymphocytic inflammatory response initially directed toward the teratoma, and subsequent systemic circulation of lymphocytes and antibodies (Figure 6).^7,8^ Lymphocytes form autoantibodies against the proteins expressed by intratumoral neural tissue, reacting against similar proteins in distant sites such as the brain and optic nerve.^7,8^ Anti-NMDAR encephalitis specifically involves autoantibodies in CSF that target the GluN1 subunit of the NMDAR.^8,10^ Histologic examination of ovarian teratomas in patients with anti-NMDAR encephalitis across multiple studies most commonly identified increased CD3 + T cell, CD4 + T cell, and CD20 + B cell lymphocyte populations.^7^

Histologic examination of the ovarian teratoma in the present case revealed neural tissue with perivascular cuffing characterized by mixed lymphocytic infiltration of predominantly CD3 + T cells with some CD79a + B cells and CD20 + B cells; a few MUM1 + plasma cells and IBA + histiocytes were present within the inflammatory foci (Figure 4). This finding correlates with the hypothesis proposed in the human medical literature that antibodies to proteins expressed by intratumoral neural tissue facilitate the immune-mediated targeting of the central nervous system, including the cerebral cortex and optic nerve.^7,8^ There are large inflammatory infiltrates surrounding intratumoral neural tissues in human cases of anti-NMDAR encephalitis.^8,13^ Nervous tissue is present in 96% of ovarian teratomas associated with anti-NMDAR encephalitis, compared to 38% of control ovarian teratomas without a corresponding PNNS; and 100% of anti-NMDAR encephalitis-associated ovarian teratomas displayed inflammatory infiltrates in contact with central nervous tissue, compared to 13% of the ovarian teratomas in patients without neurologic signs.^13^

Specific confirmatory testing in human cases requires the detection of autoantibodies in the CSF analysis or serum.^8^ Antibody titers for CSF and serum are classified as weakly positive (CSF 1:1, serum 1:10), positive (CSF 1:10 or 1:32, serum 1:32), or strongly positive (CSF 1:100 or 1:320, serum 1:100).^15^ Although autoantibody testing did not occur in this case owing to a lack of reference values, a comparison of CSF analyses between the initial and follow-up visits revealed subsequent normal CSF, and autoimmune inflammation was prioritized as the cause of the dog’s optic neuritis and meningoencephalitis. Cerebrospinal fluid analysis in dogs with immune-mediated optic neuritis is variable.^3^ In human cases of NMSOD associated with ovarian teratomas, 67% displayed mild to moderate CSF pleocytosis, consistent with the findings in this case.^21^ Additional testing commonly performed in humans includes abdominal ultrasound or CT, MRI, and electroencephalogram to determine differential diagnoses and rule out other etiologies. In one study, although 67% of patients with anti-NMDAR encephalitis had a normal brain MRI, 90% showed electroencephalogram abnormalities.^23^

Despite the severity of signs of neurologic disease and rapid disease progression in human PNNS cases, early medical immunosuppression can achieve a good clinical outcome. Surgical removal of the ovarian teratoma combined with immunosuppressive therapy has been associated with regression of signs of neurologic disease in patients with associated anti-NMDAR encephalitis, AQP4-IgG + NMSOD, or MOG-encephalomyelitis.^21–24^ Approximately 80% of patients who received this combined treatment experienced substantial improvement in neurologic disease.^11^ In the case described here, removal of the ovarian teratoma and treatment with corticosteroids resulted in cytologic remission. Though no follow-up MRI could be performed to document resolution of the cerebral lesions, the dog’s stable clinical condition years after diagnosis supports the resolution of the underlying disease. Recurrence of the PNNS in cases with improvement in neurologic disease after ovarian teratoma resection occurs less frequently compared to recurrence in patients without an associated ovarian teratoma.^7,23,24^ However, delays in tumor resection allow for the development of long-lived plasma cells and increased antibody affinity, which can prevent the resolution of neurological signs.^7^ Surgical removal and immunosuppressive therapy have also been supplemented with immunoglobulins administered intravenously and plasmapheresis in treatment of human cases.^16,23^

## Supplementary Material

aalag088_Supplemental_Table_1

## Off-label antimicrobial declaration

The authors declare no off-label use of antimicrobials.

## Institutional animal care and use committee or other approval declaration

The authors declare no institutional animal care and use committee or other approval was needed.

## Abbreviations

CSF: cerebrospinal fluid
MRI: magnetic resonance imaging
NMDAR: N-methyl-D-aspartate receptor
OD: *oculus dexter*
ON: optic neuritis
OS: *oculus sinister*
OT: ovarian teratoma
OU: *oculus uterque*
PLR: pupillary light reflex
PNNS: paraneoplastic neurological syndrome
VMTH: Veterinary Medical Teaching Hospital
