# Correction to: Optic neuritis and meningoencephalitis associated with an ovarian teratoma in a dog

**DOI:** 10.1093/jvimsj/aalag167

**Published:** 2026-07-24

This is a correction to: Hunter P Sapienza, Brian G Murphy, Kelly O’Connell, Nina Samuel, Sara A Adelman, Soohyun Kim, Karen M Vernau, Christine M Toedebusch, Adrien M Dupanloup, Optic neuritis and meningoencephalitis associated with an ovarian teratoma in a dog, *Journal of Veterinary Internal Medicine*, Volume 40, Issue 3, May–June 2026, aalag088, https://doi.org/10.1093/jvimsj/aalag088

In the originally published article, in section **Discussion**, fourth paragraph, first sentence, there was an error in a reference to a figure. The text has been corrected to read: “[...] and subsequent systemic circulation of lymphocytes and antibodies (Figure 6).” instead of: “[...] and subsequent systemic circulation of lymphocytes and antibodies (Figure 5).”

