# Supplementary material for: Optic neuritis and meningoencephalitis associated with an ovarian teratoma in a dog
Source: J Vet Intern Med. 2026 May 20;40(3):aalag088. doi: 10.1093/jvimsj/aalag088 (PMC13189267; doi:10.1093/jvimsj/aalag088)
Supplement: aalag088_Supplemental_Table_1 [file aalag088_supplemental_table_1.docx]

| **IHC Assay** | **Primary Clone** | **Manufacturer** | **Antigen Retrieval Technique** | **Secondary Antibody** | **Antibody Concentration** | **Incubation Time** |
| --- | --- | --- | --- | --- | --- | --- |
| CD3 | CD3-12 | Peter Moore Davis, CA | VMTH Citrate, pH 6.1 at 98^o^C for 30 minutes | ImmPRESS HRP Goat Anti-Rat Polymer Kit (Ref: MP-7404-50) for 30 minutes | 1:30 | Primary antibody for 1 hour |
| CD79a | HM57 | BIORAD (Ref: MCA2538H) | VMTH Citrate, pH6.1 at 98°C for 30 minutes | Mouse-on-Canine HRP-Polymer (Ref: MC541L) for 30 minutes | 1:100 | Primary antibody for 1 hour |
| CD20 | SP32 | Abcam (Ref: ab64088) | VMTH Citrate, pH6.1 at 98°C for 30 minutes | Rabbit-on-Canine HRP-Polymer (Ref: RC542L) for 30 minutes | 1:100 | Primary antibody for 1 hour |
| MUM1 | BC5 | Biocare (Ref: CRM352) | Borg Decloaker (Ref:BD1000G1) at 98°C for 30 minutes | Rabbit-on-Canine HRP-Polymer (Ref: RC542L) for 30 minutes | 1:100 | Primary antibody for 30 minutes |
| IBA1 | EPR16588 | Abcam (Ref: ab178846) | VMTH Citrate, pH6.1 at 98°C for 30 minutes | Rabbit-on-Canine HRP-Polymer (Ref: RC542L) for 30 minutes | 1:450 | Primary antibody for 1 hour |

**Supplemental Table 1:** Detailed protocols for each immunohistochemistry assay used to characterize the inflammatory populations of neural tissue within the ovarian teratoma.
